# Relationship Between Replay-Associated Ripples and Hippocampal *N*-Methyl-D-Aspartate Receptors: Preliminary Evidence From a PET-MEG Study in Schizophrenia

**DOI:** 10.1093/schizbullopen/sgac044

**Published:** 2022-07-07

**Authors:** Matthew M Nour, Katherine Beck, Yunzhe Liu, Atheeshaan Arumuham, Mattia Veronese, Oliver D Howes, Raymond J Dolan

**Affiliations:** Max Planck University College London Centre for Computational Psychiatry and Ageing Research, London WC1B 5EH, UK; Wellcome Trust Centre for Human Neuroimaging, University College London, London WC1N 3AR, UK; Department of Psychosis Studies, Institute of Psychiatry Psychology and Neuroscience, King’s College London, London SE5 8AF, UK; Department of Psychiatry, University of Oxford, Oxford OX3 7JX, UK; Department of Psychosis Studies, Institute of Psychiatry Psychology and Neuroscience, King’s College London, London SE5 8AF, UK; Max Planck University College London Centre for Computational Psychiatry and Ageing Research, London WC1B 5EH, UK; State Key Laboratory of Cognitive Neuroscience and Learning, IDG/McGovern Institute for Brain Research, Beijing Normal University, Beijing 100875, China; Chinese Institute for Brain Research, Beijing 102206, China; Department of Psychosis Studies, Institute of Psychiatry Psychology and Neuroscience, King’s College London, London SE5 8AF, UK; Department of Psychosis Studies, Institute of Psychiatry Psychology and Neuroscience, King’s College London, London SE5 8AF, UK; Department of Information Engineering, University of Padua, Padua, Italy; Department of Psychosis Studies, Institute of Psychiatry Psychology and Neuroscience, King’s College London, London SE5 8AF, UK; Max Planck University College London Centre for Computational Psychiatry and Ageing Research, London WC1B 5EH, UK; Wellcome Trust Centre for Human Neuroimaging, University College London, London WC1N 3AR, UK

**Keywords:** Psychosis, inference, sharp wave ripple, excitation-inhibition balance, replay

## Abstract

**Background and Hypotheses:**

Hippocampal replay and associated high-frequency ripple oscillations are among the best-characterized phenomena in resting brain activity. Replay/ripples support memory consolidation and relational inference, and are regulated by *N*-methyl-D-aspartate receptors (NMDARs). Schizophrenia has been associated with both replay/ripple abnormalities and NMDAR hypofunction in both clinical samples and genetic mouse models, although the relationship between these 2 facets of hippocampal function has not been tested in humans.

**Study Design:**

Here, we avail of a unique multimodal human neuroimaging data set to investigate the relationship between the availability of (intrachannel) NMDAR binding sites in hippocampus, and replay-associated ripple power, in 16 participants (7 nonclinical participants and 9 people with a diagnosis of schizophrenia, PScz). Each participant had both a [^18^F]GE-179 positron emission tomography (PET) scan (to measure NMDAR availability, *V*_*T*_) and a magnetoencephalography (MEG) scan (to measure offline neural replay and associated high-frequency ripple oscillations, using Temporally Delayed Linear Modeling).

**Study Results:**

We show a positive relationship between hippocampal NMDAR availability and replay-associated ripple power. This linkage was evident across control participants (*r*(5) = .94, *P *= .002) and PScz (*r*(7) = .70, *P* = .04), with no group difference.

**Conclusions:**

Our findings provide preliminary evidence for a relationship between hippocampal NMDAR availability and replay-associated ripple power in humans, and haverelevance for NMDAR hypofunction theories of schizophrenia.

## Introduction

Spontaneous hippocampal activity during rest (“offline”) periods is thought to play a key role in myriad cognitive processes, including memory consolidation, relational inference, and stabilization of neural representations.^1–5^ In rodents, such activity is exemplified by sequential hippocampal place cell reactivations that “replay” previous experiences, and replay-associated sharp wave ripple (SWR) oscillations (SWR *>*100 Hz). Replay/ripples have been proposed to play a role in symptom generation across multiple psychiatric disorders, from psychosis to anxiety.^6–9^ Such hypotheses have only recently begun to be tested in clinical samples, owing to analytic advances in measuring offline replay signatures in humans using noninvasive functional neuroimaging tools.^7,10,11^

Replay and ripple oscillations are exquisitely sensitive to a balance between local excitatory and inhibitory neural populations,^1,12^ and to hippocampal *N*-methyl-D-aspartate receptor (NMDAR) signaling.^4,13–17^ Cortical excitation-inhibition imbalance and NMDAR hypofunction are also conjectured to be central to the pathoetiology of schizophrenia.^18–31^ Indeed, multiple lines of pharmacological, neuroimaging, and genetic evidence implicate NMDAR hypofunction in schizophrenia. Postmortem studies in people with a diagnosis of schizophrenia (PScz) show reductions in hippocampal NMDAR binding,^32^ while NMDAR antagonists can induce acute psychotic symptoms in healthy volunteers,^25,26,31^ and reproduce neurophysiological signatures detected in PScz.^20,23^ Moreover, in vivo molecular neuroimaging studies using NMDAR radioligands ([^18^F]GE-179 positron emission tomography (PET) and [^123^I]CNS-1261 single photon emission tomography [SPET]) report reductions in hippocampal NMDAR availability in PScz.^21,30,33^

Schizophrenia is also associated with abnormalities in resting hippocampal activity (eg, hypermetabolism and hyperactivity).^34–37^ Particularly relevant are findings from genetic mouse models of schizophrenia that find abnormalities in both hippocampal replay and associated ripple oscillations during rest,^38–40^ and convergent findings from a recent study investigating analogous neural reactivation signatures using magnetoencephalography (MEG) in a clinical sample of PScz.^7^

The relationship between hippocampal NMDAR availability and replay events has yet to be examined in humans. Here we address this question in a unique sample of PScz and cognitively matched control participants. Participants completed 2 brain scans each: a MEG scan (to measure spontaneous neural replay of learned task structure during a rest session^7^) and a [^18^F]GE-179 PET scan (to measure regional availability of NMDARs^30^). We hypothesized that hippocampal NMDAR availability would correlate with the strength of offline replay and associated ripple power (previously shown to emanate from hippocampal sources^7^), in line with a key role for NMDAR-dependent synaptic plasticity in hippocampal reactivations.^15–17^ We find a positive correlation between hippocampal NMDAR availability and replay-associated ripple power (but not replay per se). This was true for both control participants and PScz, with no group difference in this relationship.

## Materials and Methods

### Participants and Data Sets

This study availed of 2 previously published data sets: MEG replay (Data set A^7^) and [^18^F]GE-179 PET (Data set B^30^). The findings presented in the present article pertain to the subset of participants who participated in both studies (*n* = 10 PScz: 3F, mean age at MEG = 26.4 years, range 20–34, 5 unmedicated [defined as no oral/depot antipsychotic medication for 6 weeks/months, respectively], and 7 healthy control participants: 1F, mean age at MEG = 28.7 years, range 22–36). We excluded 1 PScz participant from all analyses as this participant exhibited an extreme hippocampal *V*_*T*_ effect size (*>*2.5 SD from the group median). See Supplementary Materials and Methods for full inclusion/exclusion criteria and clinical/cognitive assessments. See table 1 for details of study sample.

**Table 1.** Participant Demographic, Cognitive, Clinical and PET variables. (Information From Full PET-MEG Subsample)

Variable	Control	PScz	Group Comparison^++^
Demographics			
Sample size	7	10	
Gender	1F, 6M	3F, 7M	χ ^2^ = 0.57 (*P* = .45)
Age (mean, SD)	28.7 (5.46)	26.4 (4.43)	*t* = 0.97 (*P* = .35)
Years in education (mean, SD)	20.0 (0.93)	15.90 (3.98)	*t *= 2.41 (*P* = .03)
Employment status [F/P/U]*	1/ 2/ 4	2/ 2/ 6	χ ^2^ = 3.24 (*P* = .07)
Handedness	5R, 2L	10R, 0L	χ ^2^ = 9.26 (*P* = .05)
Ethnicity [W/BAME/Other]^†^	2/ 5/ 0	2/ 7/ 1	χ ^2^ = 9.26 (*P* = .05)
Alcohol units week^−^1 (mean, SD)	9.29 (6.26)	0.10 (0.32)	*z* = 3.60 (*P* = .00)
Current cannabis (not within 1 week)	4	4	χ ^2^ = 0.49 (*P* = .49)
Current smoker (not within 6 hrs)	4	3	χ ^2^ = 0.01 (*P* = .91)
Cognitive			
IQ (SD)	105.4 (2.94)	99.7 (8.86)	*z* = 1.08 (*P* = .28)
Digit span forward (mean, SD)	6.71 (0.95)	5.75 (1.38)	*t* = 1.60 (*P* = . 13)
Digit span backward (mean, SD)	4.07 (0.93)	3.30 (0.82)	*z* = 1.69 (*P* = .09)
Psychiatric			
Depressive symptoms^‡^ (mean, SD)	0.29 (0.49)	10.10 (6.59)	*z* = −3.42 (*P < *.001)
Positive symptoms^§^ (mean, SD)	7.14 (0.38)	17.20 (7.35)	*z* = −3.09 (*P < *.001)
Negative symptoms^§^ (mean, SD)	7.00 (0.00)	19.0 (6.85)	*z* = −3.45 (*P < *.001)
General psychopathology^§^ (mean, SD)	16.14 (0.38)	29.0 (8.69)	*z* = −3.45 (*P < *.001)
General functioning^**^ (mean, SD)	97.14 (4.88)	63.2 (13.09)	*z* = 6.50 (*P < *.001)
Clinical details			
No. taking medication	—	5^¶^	—
Months since 1st symptom (median, IQR)	—	43 (36)	—
No. acute episodes (median, IQR)	—	3 (4)	—
No. admissions (median, IQR)	—	1.5 (3)	—
PET			
Injected Dose MBq (mean, SD)	139.93 (9.31)	140.68 (4.79)	*z* = 0.63 (*P* = .53)
Total motion mm (mean, SD)	8.70 (6.48)	15.10 (11.16)	*z* = −1.22 (*P* = .22)
Hippocampal volume mm^3^, bilateral (mean, SD)	8732 (635)	9066 (629)	*t *= −1.07 (*P* = .30)
Brain K1 (mean, SD)	0.28 (0.08)	0.24 (0.05)	*z* = 0.73 (*P* = .46)
Hippocampus K1 (mean, SD)	0.26 (0.06)	0.23 (0.06)	*z* = 0.53 (*P* = .53)
Time between PET and MEG, years (mean, SD)	1.61 (0.79)	1.20 (0.91)	*t* = 0.97 (*P* = .35)

*F = full-time employed, P = part-time employed, U = unemployed (inc. student).

^†^W = White. BAME = Black, Asian, and Minority Ethnic. Other (inc. multiple groups).

^‡^Montgomery Asberg Depression Rating Scale (MADRS), floor = 0.

^§^Positive and Negative Syndrome Scale (PANSS) scale, floor = 7 (pos), 7 (neg), 16 (gen).

^**^General Assessment of Functioning (GAF) scored from 0 to 100.

^¶^D2/3 receptor antagonist medication per medicated PScz: (1) aripiprazole 10 mg day^−1^, (2) lurasidone 37 mg day^−1^, (3) aripiprazole 400 mg month^−1^ (depot), (4) olanzapine 7.5 mg day^−1^, (5) paliperidone 50 mg month^−1^ (depot).

^++^Group comparisons: For continuous variables, unpaired *t* test (*t*) or Wilcoxon rank sum test (z) (for non-normally distributed data). For categorical variables, Chi squared test (χ ^2^). Two-tailed hypotheses. SD: standard deviation. IQR: interquartile range. Time-sensitive variables (eg, age, symptom/cognitive scores) recorded at MEG. PScz: people with a diagnosis of schizophrenia. K1 reflects the rate constant for transfer from arterial plasma to tissue (mL cm^−3^ min^−1^).

### MEG Applied Learning Task

**Fig. 1.**
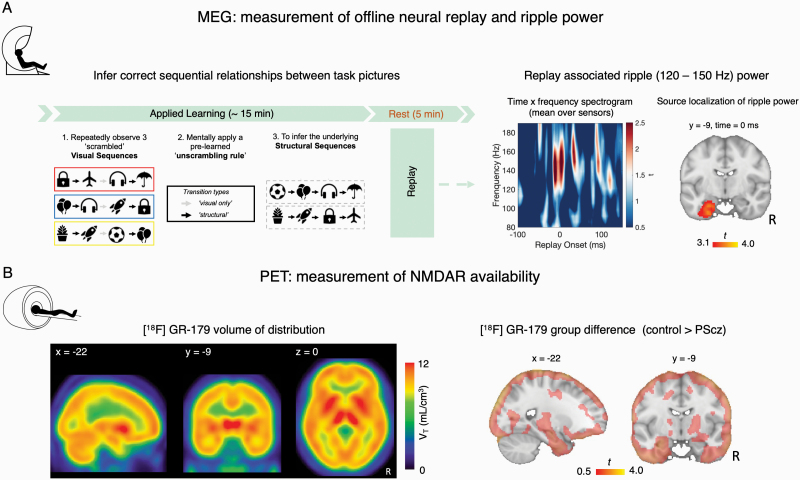
Quantifying replay MEG and NMDAR availability PET. **(A)** (Left) Applied Learning MEG task.^7^ During MEG participants needed to infer the sequential relationships (“structural sequences”) between 8 task pictures, from scrambled “visual sequences” containing these pictures. To do this they needed to leverage the knowledge of how visual sequences mapped to structural sequences (“unscrambling rule”), which was learned prior to MEG (see “MEG Applied Learning Task”). The Applied Learning task was followed by a 5-minute awake rest session. (Right) In MEG data from this postlearning rest session, we tested for the presence of spontaneous neural replay of correctly inferred task transitions using a decoding-based analytic approach. In Nour et al.,^7^ we identified transient increases in high frequency “ripple” power (120–150 Hz) coincident with replay onsets. Spectrogram shows the average high-frequency power increase at putative replay onset, averaged over all putative replay events, MEG sensors, and participants (*n *= 53 [27 controls, 26 PScz], the MEG sample reported in Nour et al.^7^) (plotted as *t*-statistic of 1-sample *t* test, 2-tailed). 0 ms represents time of thresholded replay event (time samples exceeding the subject-specific 95th percentile for replay evidence, preceded by a low-reactivation baseline, see “Materials and Methods” and in Nour et al.^7^). We further source localized repay-associated ripple power increases to hippocampus. Significant source localization cluster of replay-associated ripple power reproduced from Nour et al.^7^ (*n* = 53 [27 controls, 26 PScz], significance at whole-brain *P*_FWE_ < .05, cluster-based permutation test, 5000 permutations, cluster-defining threshold *t* > 3. **(B)** (Left) *N*-methyl-D-aspartate receptor (NMDAR) availability was estimated for each participant using [^18^F]GE-179 PET. Mean [^18^F]GE-179 volume of distribution (*V*_*T*_) at each voxel, indexing availability of open NMDARs (ie, intrachannel binding sites) (mean over *n* = 16 participants, 9 PScz, and 7 controls). (Right) *t* values of the group difference (control *> *PScz) in mean *V*_*T*_ estimate at each voxel. Control participants exhibit numerically greater mean *V*_*T*_ estimates throughout the cortex compared to PScz, but this group difference is not statistically significant. Note very liberal image thresholding at t *> *0.50, and excluding cerebellum, for illustration purposes only. For all images: neurological orientation, and MNI coordinates of section as given.

During MEG, participants performed a validated relational inference (“Applied Learning”) task, previously shown to elicit neural replay during a post-task rest session.^7,10^ During the task participants needed to infer the sequential relationships between 8 task pictures, where these relationships formed 2 (“structural”) sequences ([*A* → *B* → *C* → *D*] & [*A*′ → *B*′ → *C*′ → *D*′]).^10^ Crucially, participants were never shown these complete sequences, and instead needed to infer the correct sequential relationships by passively observing scrambled “visual sequences” containing task pictures, and applying an “unscrambling rule,” learned before MEG (figure 1A). See Nour et al.^7^ for full details, including behavioral data indicating no difference between PScz and controls in the acquisition of task structural knowledge at the end of the Applied Learning task.

MEG contained 2 additional sessions of relevance. First, a Stimulus Localizer task prior to Applied Learning, wherein we presented each task picture in a random order (1 s presentation, ~40–52 presentations per picture), to obtain visually evoked MEG data for training stimulus decoders. Second, a 5-minute eyes-open rest session immediately after Applied Learning, wherein we quantified spontaneous neural replay of inferred task sequences (see “MEG Sequenceness Analysis”, below).

### MEG Acquisition and Preprocessing

As previously described,^7 ^MEG was recorded continuously at 1200 samples/second using a whole-head 275-channel axial gradiometer system (CTF Omega, VSM MedTech), while participants sat upright (3 sensors not recorded due to excessive noise in routine testing). Sensor data were high-pass filtered at 0.5 Hz using a first-order IIR filter, and downsampled to 100 Hz (sequenceness analysis) and 400 Hz (time-frequency analysis). Excessively noisy data segments and sensors were automatically identified and removed from the data. Independent Component Analysis (FastICA, http://research.ics.aalto.fi/ica/fastica) was used to decompose the sensor data for each session into 150 temporally independent components and associated sensor topographies. Artifact components were classified by automated inspection of the spatial topography, time course, kurtosis of the time course, and frequency spectrum, and subtracted from the data.

### MEG Sequenceness Analysis

Full details of MEG analysis are provided in Supplementary Materials and Methods and Nour et al.^7 ^ Sequenceness analysis relies on the ability to decode transient spontaneous neural reactivations of task stimulus representations from MEG data collected during resting state. First, we characterized participant-specific MEG sensor patterns corresponding to each task picture using visually evoked MEG patterns from a pre-learning Stimulus Localizer task. As previously described,^7^ for each task picture (*n *= 8) we trained a separate one-vs-rest lasso-regularized logistic regression (decoding) model using epoched MEG sensor-level data from Stimulus Localizer, and assessed prediction accuracy for the family of trained decoders at each time point of the visually evoked response in leave-one-out cross-validation. Group-level cross-validated peak decoding accuracy was at 180 ms after picture onset. See Nour et al.^7^ for full decoding accuracy assessment, including demonstration of no significant difference between PScz and control participants.

We then applied trained decoders (from the peak accuracy time bin) to MEG (sensor-level) data from each time point of the post-learning rest session to generate a [time, state] reactivation probability matrix, and used a Temporally Delayed Linear Modeling framework to quantify evidence for sequential reactivations consistent with the inferred task transition structure.^11^

In our previous work,^7^ we found maximal evidence for spontaneous neural replay at 40 ms state → state transition lag (ie, neural reactivation of state A followed by reactivation of state B, 40 ms later). In the present work we therefore identified time points during the rest session where strong reactivation of 1 stimulus (eg, A) was followed by strong reactivation of another stimulus that is adjacent in the learned task sequence (eg, B), with 40 ms lag. We identified replay events that were preceded by a pre-event baseline of low replay probability (see Supplementary Materials and Methods). We epoched the post-learning rest MEG data surrounding each such putative replay event. For each epoch (event) we then computed a frequency decomposition (wavelet transformation) in the window −100 to + 150 ms with respect to replay onset, for each (non-artefactual) sensor. Averaging this estimate over sensors and epochs resulted in a [time, frequency] matrix for each participant, capturing the typical spectrally resolved power change at replay onset. For each participant, we then extracted the mean power change at replay onset in the previously identified spectral region of interest (ROI) (120–150 Hz)^7,10^ (figure 1A).

Finally, we conducted a beamforming (source localization) analysis on the epoched MEG data to identify putative intracranial sources correlating with increased ripple power (120–150 Hz) at replay onset, as in Nour et al.,^7^ using a linearly constrained minimum variance (LCMV) beamforming algorithm^41^ (figure 1A).

### PET Image Acquisition

[^18^F]GE-179 PET was conducted with a Siemens 3T Biograph mMR PET/MR scanner (Siemens), as part of a larger study published in Beck et al.^30^ As described in this former study, PET scans started with a bolus injection of [^18^F]GE-179 (see table 1 for mean injected activity), followed by 90 minutes of continuous simultaneous PET-MR acquisition. Arterial blood samples were collected from all participants (https://www.swisstrace.ch/), and used for kinetic modeling (continuous sampling from 0 to 16 minutes, followed by 6 discrete samples, from cannula inserted into radial artery). We also acquired a T1-weighted structural MRI scan (Magnetization Prepared Rapid Gradient-Echo image) for image coregistration, and a separate low dose CT scan (140 kV, 10 mA, helical acquisition, GE Discovery DST 710 PET/CT, GE Healthcare) for tissue attenuation correction during PET image reconstruction.^30^

### PET Kinetic Modeling

Full details of PET analysis are provided in Supplementary Materials and Methods. As described in Beck et al.,^30^ prior to kinetic modeling, all PET scans underwent the same image processing pipeline to correct for head motion, segment brain tissues, and extract [^18^F]GE-179 tracer activity. NMDAR availability was operationalized as the [^18^F]GE-179 volume of distribution (*V*_*T*_).

*V*_*T*_ was estimated in 2 ways, which were highly correlated.^30^ In a ROI analysis a single *V*_*T*_ measure was derived from [^18^F]GE-179 activity within a single hippocampal mask (eg, bilateral hippocampus as defined by a probabilistic neuroanatomical atlas^42^). For this ROI analysis *V*_*T*_ was estimated using a 2-tissue compartment modeling method with metabolite-corrected arterial plasma input function.^30,43–45^ *V*_*T*_ was also estimated at the individual voxel level, using the Logan graphical approach.^46^

### Statistical Analysis

We tested for linear relationships between hippocampal NMDAR availability ([^18^F]GE-179 *V*_*T*_, from PET) and replay-associated ripple power (where the latter is defined as mean power change in 120–150 Hz range at time of putative replay onset, as defined above, from MEG). Each participant contributed a single pair of MEG-PET measurements. We also report ROI results when using a measure of “peak” ripple power increase within a replay epoch, as this measure was also reported in Nour et al.^7^

**Fig. 2.**
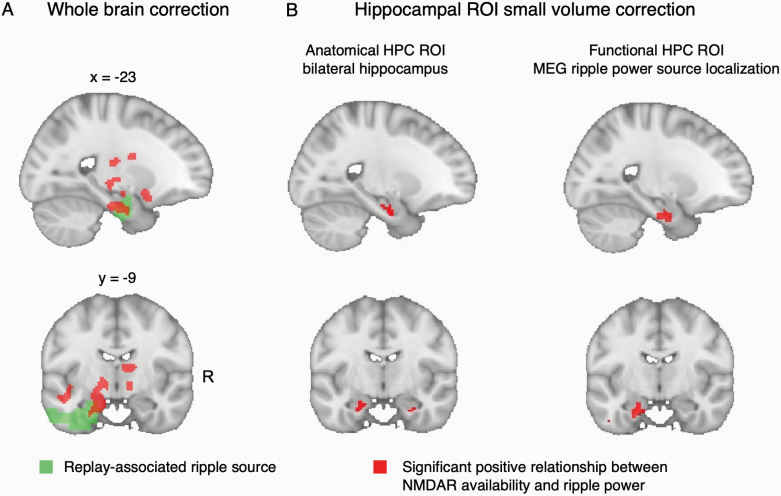
Relationship between *N*-methyl-D-aspartate receptor (NMDAR) availability and ripple power: voxelwise analysis. **(A)** *(Red)* Voxel clusters exhibiting a significant linear relationship between [^18^F]GE-179 *V*_*T*_ and replay-associated ripple power, family-wise error corrected at whole-brain cluster level (cluster-level *P*_FWE_ *< *.05 (cluster-defining threshold [CDT] *P* *< *.001 (uncorrected), critical cluster size = 747). Effect is derived from the combined sample of PScz and controls, using a multiple regression analysis (at each voxel) regressing [^18^F]GE-179 *V*_*T*_ onto replay-associated ripple power, controlling for group differences in mean [^18^F]GE-179 *V*_*T*_ and group * ripple interaction. Replay-associated ripple power is defined as subject-specific ripple power detected at replay onsets during a post-learning rest session in MEG (mean 120–150 Hz power increase at replay onset, measured overall magnetoencephalography (MEG) sensors, compared to a pre-onset baseline, as in Nour et al.^7^ (*Green*) Putative intracranial source of replay-associated ripple power, identified from previously published beamforming analysis (whole-brain cluster-level significance *P*_FWE_ *< *.05, 5000 permutations, CDT *t* *> *3.^7^ Beamforming result reproduced in figure 1A). **(B)** Small volume correction (SVC) analysis of the voxelwise linear association between [^18^F]GE-179 *V*_*T*_ and replay-associated ripple power (shown in (A)), thresholded at *P*_SVC FWE_ *< *.05 (voxel level). (Left) Small-volume ROI is bilateral hippocampal (HPC) anatomical mask, showing bilateral significant peak effects: Left peak MNI = [−16, −14, −22], peak-level *P*_FWE_ = .012. Right peak MNI = [28, 10, −28], peak-level *P*_FWE_ = .028. (Right) Small-volume ROI is the left hippocampal (HPC) cluster identified in the previously published beamforming analysis of replay-associated ripple power (green cluster in (A), “functional” mask). Peak-level effect: Left peak MNI = [−16, −14, −26], peak-level *P*_FWE_ = 0.008. For all images: Neurological orientation, MNI coordinates of view [x = −23, y = −9]. Sample: *n* = 9 PScz, *n *= 7 controls.

We first used a voxelwise multiple regression analysis at the group level, regressing the [^18^F]GE-179 *V*_*T*_ brain images onto a design matrix comprising 4 predictor variables: (1) group indicator variable (effects coded), (2) replay-associated ripple power in PScz (entries for control participants set to 0), (3) replay-associated ripple power in controls (entries for PScz set to 0), and (4) a constant term. The replay-associated ripple power variable was mean-centered across participants prior to regression. This approach allowed us to define statistical contrasts for the mean linear PET × MEG relationship (ie, slope) across groups ((2) + (3), corresponding to the primary results of this article in figure 2), the difference in such an effect between groups ((2) − (3) or (3) − (2) subtraction contrasts), and the difference in mean [^18^F]GE-179 *V*_*T*_ between groups (regressor (1), ensuring that group differences in mean *V*_*T*_ do not confound our estimate of group differences in the *V*_*T*_-ripple slope).

For voxelwise results we assess for family-wise error (FWE) corrected statistical significance both at whole-brain cluster-level *P*_FWE_ *< *.05 (cluster-defining threshold *P* *< *.001 [uncorrected]) and at voxel (peak) level (following small volume correction [SVC] for hippocampal ROIs) using random field theory, as implemented in the Statistical Parametric Mapping 12 MATLAB toolbox (SPM12, https://www.fil.ion.ucl.ac.uk/spm/software/spm12/). We use 2 hippocampal ROIs in the SVC analysis: an “anatomical” ROI corresponding to a bilateral hippocampal mask from a probabilistic neuroanatomical atlas,^42^ and a “functional” ROI corresponding to the left hippocampal cluster that exhibited a significant positive association with replay-associated ripple power at the group level, in our previous beamforming analysis (reproduced in figure 1A^7^).

In addition to the voxelwise analysis, we also conduct an ROI analysis of the linear relationship between [^18^F]GE-179 *V*_*T*_ (extracted from the bilateral anatomical hippocampal mask, as described in the above description of PET kinetic modeling) and “replay-associated ripple power.” We compute the correlation coefficient for this relationship for PScz and control participants separately, in addition to testing for a difference in the slope of the relationship between groups using a multiple regression approach:


$$\begin{array}{c} ripple=\beta_{0}+\beta_{1}*group+\beta_{2}*V_{T}+\beta_{3}*(group*V_{T}) \end{array}$$

The [^18^F]GE-179 *V*_*T*_ variable was mean-centered across participants prior to regression. Group was effects coded (PScz = −0.5, controls = +0.5).

When considering single variable effects or bivariate correlations, we conducted a formal test that the effects in question were sampled from a population with a normal distribution (Shapiro–Wilk test) prior to using parametric tests (eg, unpaired *t* test, Pearson’s correlation), and used nonparametric equivalent tests where this null hypothesis was rejected at α* *= 0.05 (eg, Wilcoxon rank sum test for equal medians, correlation and regression analyses conducted on rank-ordered variables). For all analyses, summary effects are reported as mean ± 1 standard error of the mean (SEM), and we consider (FWE-corrected) *P* *< *.05 (2-tailed) as statistically significant, unless otherwise stated. For software details, see Supplementary Materials and Methods.

## Results

### Quantifying NMDAR Availability and Ripple Power in the Same Participants

In the present study, we revisit 2 previously published data sets. The first comprises an MEG data set from Nour et al.,^7^ where participants were tasked to infer the correct sequential relationships (“structural sequences”) between 8 task pictures, before completing a 5-minute awake rest session (figure 1A). Here, using a decoding-based analytic approach, we found evidence for spontaneous neural replay of inferred sequences in MEG data from the post-learning rest session, and showed that these replay events coincided with a transient increase in high frequency (“ripple,” 120–150 Hz) power emanating from hippocampal sources (figure 1A), as in Liu et al.^10^ We also showed that PScz exhibited disruptions in both replay and ripples, which related to behavioral signatures of inferential reasoning and neural representations of the learned task structure.^7^

The second data set used PET in conjunction with the NMDAR ligand [^18^F]GE-179 to index brain NMDAR availability.^30^ In this study, PScz exhibited a reduction in hippocampal NMDAR availability relative to control participants.^30^ The present analysis involves the subset of 16 participants who consented to take part in both a MEG and PET scan (*n* = 9 PScz [4 unmedicated] and *n* = 7 control participants, see table 1).

NMDAR availability was quantified as [^18^F]GE-179 total volume of distribution (*V*_*T*_).^43,45^ As GE-179 binds to the NMDAR phencyclidine/ketamine intrachannel binding site, [^18^F]GE-179 *V*_*T*_ reflects the regional availability of open (“active”) NMDARs.^30^ In the PET-MEG subsample, [^18^F]GE-179 *V*_*T*_ was pronounced across cortical and subcortical areas, including the hippocampal cortex (figure 1B). Although mean [^18^F]GE-179 *V*_*T*_ was numerically larger in control participants compared to PScz across widespread cortical regions, this difference did not surpass FWE-corrected significance criteria in any voxels either at whole-brain level, or in a SVC analyses focusing on hippocampus (figure 1B). This lack of significance might reflect the small size of the PET-MEG subsample.^21,30^

### Relationship Between NMDAR Availability and Ripple Power: Voxelwise Analysis

We tested for a linear association between [^18^F]GE-179 *V*_*T*_ and replay-associated ripple power at the group-level (across participants). A secondary hypothesis was that there would be a difference in the slope of this relationship between PScz and controls, though our sample size limited our power to reliably assess this. Thus, we first implemented a whole-brain group-level multiple regression analysis, regressing the voxelwise [^18^F]GE-179 *V*_*T*_ images across participants onto a predictor variable capturing our MEG-derived ripple power variable of interest. Specifically, this predictor variable was the mean increase in ripple power (120–150 Hz) at time points exhibiting maximal evidence of spontaneous replay of inferred task structure (“replay onsets”), during a post-learning rest period (ie, mean ripple power increase at 0 ms in figure 1A). In a beamforming analysis, we previously reported that this same measure of replay-associated ripple power related to an activity source emanating from left hippocampus (figure 1A, reproduced as the green cluster ROI in figure 2).

This multimodal whole-brain analysis revealed a significant linear relationship between NMDAR availability ([^18^F]GE-179 *V*_*T*_) and replay-associated ripple power in the combined sample of PScz and controls. This effect exceeded a cluster-level significance threshold in a cluster of voxels encompassing left medial temporal lobe (figure 2A), at a locus that overlapped with the likely intracranial source of the ripple power signal itself (figure 1A). Of note, although we included both PScz and control participants in this multiple regression analysis, we controlled for a difference in mean voxelwise [^18^F]GE-179 *V*_*T*_ between PScz and controls, and modeled the linear relationship between voxelwise [^18^F]GE-179 *V*_*T*_ and ripple power separately for each group so as to account for potential interaction effects (see “Materials and Methods”).

Given our a priori anatomical focus, we also conducted 2 additional SVC analyses focusing on hippocampus. The first analysis used a bilateral anatomical hippocampal region of interest (ROI).^42^ This revealed that the strength of a linear association between [^18^F]GE-179 *V*_*T*_ and ripple power was statistically significant in both left and right hippocampus at a voxel-level significance threshold (left peak MNI: [−16, −14, −22], peak-level *P*_FWE_ = .012, right peak MNI: [28, 10, −28], peak-level *P*_FWE_ = .028). The second analysis made use of a functional hippocampal mask from our previous replay analysis,^7^ where this mask corresponds to a putative intracranial source of the replay-conditional ripple power signal (ie, the same variable as used in the present second-level voxelwise regression, where this functional ROI mask is identical to the cluster in figure 1A). This analysis again revealed a significant linear association effect exceeding a voxel-level significance threshold (peak-level effect: MNI: [−16, −14, −26], peak-level *P*_FWE_ = .008, figure 2B).

We next tested whether PScz and controls exhibited different slopes in the linear relationship between [^18^F]GE-179 *V*_*T*_ and ripple power. We found no voxels or clusters that surpassed whole-brain or SVC statistical significance thresholds in this group * ripple interaction analysis. This is consistent with the notion that the relationship between NMDAR availability and replay-associated ripple power is similar in PScz and controls.

### Relationship Between NMDAR Availability and Ripple Power: Hippocampal ROI Analysis

**Fig. 3.**
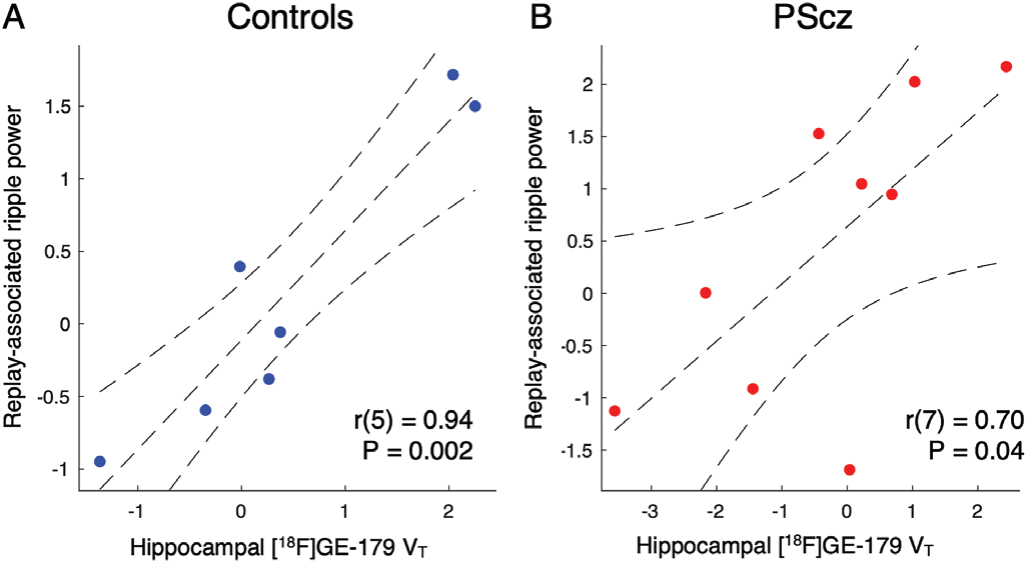
Relationship between *N*-methyl-D-aspartate receptor (NMDAR) availability ([^18^F]GE-179 *V*_*T*_) and ripple power: Hippocampal region of interest (ROI) analysis. Hippocampal [^18^F]GE-179 *V*_*T*_ estimate derived from bilateral anatomical hippocampal ROI (as in figure 2B, left). (**A)** Control participants. **(B)** People with a diagnosis of schizophrenia (PScz). Statistics are from Pearson’s correlation coefficient. Sample: *n* = 9 PScz, *n* = 7 controls.

To probe the robustness of the above findings we conducted an ROI analysis in which we derived a single hippocampal [^18^F]GE-179 *V*_*T*_ estimate for each participant, using [^18^F]GE-179 activity pooled over bilateral hippocampal voxels (not to be confused with the above analysis, in which we conducted a SVC statistical analysis following voxelwise regression). The increased signal-to-noise ratio of PET ROI analyses, as compared to that conducted at the individual voxel level, is thought to yield more robust *V*_*T*_ estimates. Consistent with the voxelwise results, we found a significant linear correlation between hippocampal [^18^F]GE-179 *V*_*T*_ and replay-associated ripple power in both controls (*r*(5) = .94, *P* = .002, Pearson’s correlation, figure 3A) and PScz (*r*(7) = .70, *P* = .04, Pearson’s correlation, figure 3B), with no group difference in the slope of this relationship (*ripple ~ group + V*_*T*_ *+ interaction* multiple regression: β _VT*group_ = 0.21 ± 0.33, *t*(12) = 0.63, *P* = .54). Of note, this positive relationship was also present across all participants when defining ripple power for each participant as the peak power increase in the frequency range 120–150 Hz, from 0 to 50 ms following a replay event onset (± 10 ms)^7^ (*ripple* ~ *group + V*_*T*_ *+ interaction* multiple regression: β _*VT*_ = 0.32 ± 0.12, *t*(12) = 2.69, *P* = .02, β _group_ = −0.77 ± 0.34, *t*(12) = −2.28, *P* = .04, β _VT*group_ = 0.18 ± 0.24, *t*(12) = 0.74, *P* = .47).

Previous studies of regional NMDAR availability in PScz have additionally used a measure of [^18^F]GE-179 binding normalized for participant-specific whole-brain *V*_*T*_ (ie, distribution of volume ratio, DVR^21,30^). Applying this approach, we find a non-significant positive relationship between replay-associated ripple power and hippocampal [^18^F]GE-179 DVR (controls: rho(5) = 0.64, *P* = .14. PScz: rho(7) = 0.20, *P* = .61, Spearman’s rank correlation).

### Relationship Between NMDAR Availability and Replay (sequenceness): Hippocampal ROI Analysis

In contrast to the observed linear relationship between hippocampal NMDAR availability and replay-associated ripple power, using a similar bilateral hippocampal ROI analysis we find no relationship between NMDAR availability and replay per se during postlearning rest in either controls (*r*(5) = −.50, *P* = .25, Pearson’s correlation) or PScz (*r*(7) = −.12, *P* = .76, Pearson’s correlation), where replay is defined as sequenceness at a 40 ms lag (as in Nour et al.^7^).

### Relationship to Potential Confounding Variables

We found no significant correlation between hippocampal NMDAR availability (V_T_ extracted from bilateral hippocampal ROI) and age (rho(14) = 0.14, *P* = .61, Spearman’s rank correlation, 2-tailed) or weekly alcohol consumption (rho(14) = 0.13, *P* = .64, Spearman’s rank correlation, 2-tailed in the combined sample of patients and controls. There was also no significant difference in median hippocampal NMDAR availability between male and female participants (ranksum = 61, *P* = .92, Wilcoxon rank sum test for equal medians, 2-tailed), nor between participants who did and did not use cannabis recreationally (at time of MEG) (ranksum = 114, *P* = .17, Wilcoxon rank sum test for equal medians, 2-tailed). Similarly, we found no association between these variables and mean ripple power at replay onset (Age: rho(14) = 0.008, *P* = .97, Spearman’s rank correlation, 2-tailed; Alcohol: rho(14) = −0.20, *P* = .45, Spearman’s rank correlation, 2-tailed; Gender: ranksum = 99, *P* = .77, Wilcoxon rank sum test for equal medians, 2-tailed; Cannabis: ranksum = 58, *P* = .92, Wilcoxon rank sum test for equal medians, 2-tailed).

## Discussion

Using a combined PET-MEG methodology, we find a linear relationship between hippocampal NMDAR availability and expression of ripple power (120–150 Hz), where the latter is time-locked to the spontaneous neural replay of a learned task structure during rest. We interpret this latter MEG signature as an analog of SWR complexes detected in rodent hippocampus.^1,7,10^ The observed relationship was present in both control participants and people with a diagnosis of schizophrenia (PScz), with no group difference. This indicates that the relationship might relate to a conserved circuit-level mechanism regulating processes such as offline human memory reactivations.

There are at least 2 mechanisms through which NMDAR signaling might impact the stability of hippocampal representations and coordination of neural state reactivations. The first relates to the function of NMDAR signaling in hippocampal plasticity, which is necessary both for stabilization of new hippocampal representations^14,15^ and learning (encoding) new associations (a prerequisite for subsequent offline replay in the context of SWRs).^4,13,15–17^ Of note, hippocampal NMDAR currents are not necessary for the retrieval of already encoded sequential associations,^17^ nor the expression of SWRs per se.^1,13,16,47–52^

Our central finding of a positive relationship between hippocampal NMDAR availability and replay-associated SWR power, seen in both PScz and controls, is consistent with this proposed role of hippocampal NMDARs in memory encoding. However, as we find no similar relationship between hippocampal NMDAR availability and replay per se, a role in encoding new sequential episodes is unlikely to be the sole basis for our results. A second mechanism pertains to the role of NMDARs in tuning the balance between excitation and inhibition at a local circuit level, given the sensitivity of place cell reactivations (ie, replay) and SWRs to hippocampal excitation-inhibition balance.^1,12,53^

The present PET-MEG results have potential relevance for neurobiological theories of schizophrenia, a disorder associated with abnormalities in both “offline” hippocampal neural activity^7,34,36,38–40^ and hippocampal NMDAR hypofunction.^21,30,32^ Genetic mouse models of schizophrenia show reduced temporal coordination in place cell reactivations (replay) and exuberant SWRs during rest (offline) periods.^38–40^ Convergent with these findings, we recently used human MEG to show that schizophrenia is linked to an exuberance in hippocampal replay-associated ripple oscillations and impaired replay, which is related to behavioral and neural signatures of structural inference.^7^ More broadly, cortical excitation-inhibition imbalance is considered a key feature of schizophrenia,^19^ evidenced in abnormalities of neural oscillations and stimulus-evoked M/EEG potentials.^18,22,54–61^ Although the molecular basis for this excitation-inhibition imbalance is incompletely understood, a leading hypothesis implicates a primary reduction in NMDAR signaling in cortical pyramidal neurons.^18,19^

The complex relationship between NMDAR signaling, cortical excitability, and replay/SWRs precludes a simple synthesis of the relevant empirical findings in schizophrenia. For example, given evidence for hippocampal NMDAR hypofunction in PScz,^18,21,30^ and the necessary role of NMDAR-dependent plasticity in replay/SWRs,^4,13,15,17^ it might appear puzzling that clinical and preclinical findings indicate *augmented* SWR power (and impaired replay) in this condition.^7,38–40^ Such seemingly paradoxical patterns might reflect allostatic mechanisms that regulate cortical excitability.^18,19,62^ For example, NMDAR hypofunction on excitatory pyramidal neurons is expected to result in reduced pyramidal neuron activity, which might trigger allostatic down-regulation of inhibitory interneurons.^18,28^ Here, the primary abnormality is expected to result in reduced replay (owing to reduced NMDAR-dependent plasticity), and the secondary changes, while not rescuing replay, may nevertheless predispose to cortical hyperexcitability (eg, augmented SWRs^7,38–40^ and resting hypermetabolism^34,36^). We acknowledge that such a proposed framework goes beyond currently available data, but we consider that it warrants further investigation both in preclinical mechanistic models, and longitudinal clinical studies investigating individuals from before symptom onset.^19^ Furthermore, it is important to note that primary NMDAR hypofunction on interneurons (as opposed to pyramidal neurons) has also been proposed in PScz.^24,35,63,64^

### Limitations of the Study

One immediate limitation of the present study is the small sample size, which reduces our power to detect group differences in the slope of the NMDAR-ripple relationship, and subtle associations between NMDAR availability and replay strength. Furthermore, larger samples are required to robustly test associations between potential confounding variables (eg, gender, alcohol, and cannabis use) and both NMDAR availability and/or MEG ripple power. This limitation reflects the challenges associated with obtaining multimodal imaging data in clinical samples.

Secondly, studies combining pharmacological interventions and MEG (eg, ketamine studies in human participants) are required to robustly test causal claims pertaining to the role of NMDAR signaling in associative memory encoding and replay in humans, and the degree to which such effects relate to induced psychotic symptoms.

A final limitation relates to the specificity of GE-179 for NMDARs from binding competition studies, compared to its nonspecific binding profile,^65–68^ particularly as *V*_*T*_ does not distinguish between non-specific and specific binding^45^ (see “PET Kinetic Modeling”). GE-179 PET binds to the intrachannel portion of the active NMDAR (the phencyclidine/ketamine binding site), such that measures of radioligand binding are thought to reflect the regional availability of “active” (ie, open) channels.^21,30,33,44,67,68^ Consistent with this claim, in a recent rat seizure model PET study, hippocampal [^18^F]GE-179 *V*_*T*_ was increased following focal electrical stimulation, an effect that was blocked by ketamine preadministration.^67^ However, other preclinical findings pertaining to the target specificity of GE-179 have been mixed.^66,68^ Such mixed findings might relate to the fact that the specificity of [^18^F]GE-179 PET binding for open NMDAR channels is itself a function of baseline NMDAR activity (closely related to neuronal depolarization), and this activity is reduced by some general anesthetics used in preclinical studies.^65,67^ Relatedly, variation in [^18^F]GE-179 binding between participants might relate to a reduction in active NMDARs, reduced NMDAR number, or increased NMDAR internalization.^30^ These latter considerations complicate the interpretation of our reported association between NMDAR “availability” and a marker of hippocampal neuronal activity (ripple power).

## Conclusions

In summary, we provide preliminary evidence for a relationship between hippocampal NMDAR availability and replay-associated ripple power in humans. Our findings add to evidence that NMDARs play an important regulatory role in offline hippocampal activity, and motivate future studies that probe the nature of this relationship in schizophrenia.

## Supplementary Material

sgac044_suppl_Supplementary_Material
