# Supplementary material for: Relationship Between Replay-Associated Ripples and Hippocampal N-Methyl-D-Aspartate Receptors: Preliminary Evidence From a PET-MEG Study in Schizophrenia
Source: Schizophr Bull Open. 2022 Jul 7;3(1):sgac044. doi: 10.1093/schizbullopen/sgac044 (PMC9334566; doi:10.1093/schizbullopen/sgac044)
Supplement: sgac044_suppl_Supplementary_Material [file sgac044_suppl_Supplementary_Material.docx]

# Supplementary Materials and Methods

### **Participants and datasets**

This study availed of two previously published datasets: MEG replay (Dataset A, ^1^) and [^18^F]GE-179 PET (Dataset B, ^2^). The studies were approved by the London Westminster NHS Research Ethics Committee (15/LO/1361), the West London & GTAC Research Ethics Committee (16/LO/0130) and the Administration of Radioactive Substances Advisory Committee. All participants provided written, informed consent to participate in both studies, and were compensated for their time. Participants who had completed a PET scan under the study of Dataset B were invited to complete an MEG scan under the study of Dataset A.

PScz were recruited from London community psychosis NHS clinics, and diagnosis confirmed with the Structured Clinical Interview for DSM-IV-TR, Axis I Disorders, SCID-I ^3^. Control participants were recruited from the same geographical area through online advertisements. The combined general exclusion criteria over both datasets were age *>* 45 years, poor vision limiting performance, not having been educated in English, history of significant head trauma resulting in loss of consciousness for *>*1 minute, contraindication to PET or MR imaging (e.g., pregnancy, ferromagnetic implants), or current medication that might affect NMDAR function (i.e., no use within 3 months of PET of antidepressants, mood stabilizers or benzodiazepines). Healthy volunteers were not taking neurological or psychiatric medication, had no history of neurological or psychiatric disorder (assessed by SCID-I ^3^), and no family history of psychosis in a first degree relative. PScz had no neurological or psychiatric comorbidity. All participants had a negative urine drug screen for stimulants (SureScreen Diagnostics, Derby, UK) on PET scan day ^2^.

Prior to MEG, we assessed psychiatric symptoms with the Positive and Negative Syndrome Scale (PANSS) scale ^4^, Montgomery ˚Asberg Depression Rating Scale (MADRS)^5^, and General Assessment of Function (GAF) ^6^, and also administered brief measures of IQ (the Wechsler Test of Adult Reading, WTAR ^7^) and working memory (mean of forward and backward Digit Span). See **Table 1**.

### **MEG sequenceness analysis**

Sequenceness analysis relies on the ability to quantify evidence for transient spontaneous neural reactivations of task stimuli from MEG sensor patterns during rest. For each task picture (n = 8) we trained a separate one-vs-rest lasso-regularized logistic regression model using epoched MEG sensor-level data from Stimulus Localizer, and assessed prediction accuracy for the family of trained decoders at each time point of the visually-evoked response in leave-one-out cross validation. Group-level cross-validated peak decoding accuracy was at 180 ms after picture onset. Decoders from this maximal-accuracy training time bin were used for sequenceness analysis ^8–10^. See Nour et al., (2021) for full decoding accuracy assessment, including demonstration of no significant difference between PScz and control participants.

We applied trained decoders (from the peak accuracy time bin) to MEG (sensor-level) data from each time point of the post-learning rest session to generate a [time, state] reactivation probability matrix, and used a Temporally Delayed Linear Modelling (TDLM) framework to quantify evidence for sequential reactivations consistent with the inferred task transition structure ^11^. TDLM is a multiple linear regression approach that quantifies the degree to which a lagged (i.e., past) reactivation time course of state *i*, (*X*(∆*t*)*_i_*, *t* indicates lag time) can predict the reactivation time-course of state *j*, (*X_j_*) ^9–11^.

As described in Nour et al., (2021), we performed a separate (first-level) family of multiple regressions using each state’s (*j* ∈{1 : 8}) reactivation time course as a dependent variable, and the historical (i.e., time-lagged) reactivation time courses of all states (*i* ∈{1 : 8}) as predictor variables:

$$\begin{aligned} X_{j}= \sum_{i=1}^{8} {\beta\left( \Delta t \right)}_{i,j}+C \end{aligned}$$

The predictor (design) matrix from a single model contained a separate predictor for the reactivation time courses of all states (*i* ∈{1 : 8}), lagged by ∆*t* ∈{10*ms,*20*ms,...,*100*ms*}, plus the reactivation time course of all states lagged by ∆*t*+*α*, where *α* = [100*ms,*200*ms,...*500*ms*], to capture autocorrelations in state time courses at a canonical alpha frequency, in addition to a constant term, C. We repeat the regression from the equation above for each *j* ∈ {1 : 8} and ∆*t* ∈{10*,* 20*,* 30*, ...,* 100 *ms*}, and use ordinary least squares regression to obtain *β*.

The regression coefficients from the equation above quantify the evidence for each empirical state → state reactivation pattern at a specific lag, ∆*t*. For example, *β*(∆*t*)*_i,j_* is the coefficient capturing the unique variance in *X_j_* explained by *X*(∆*t*)*_i_*. All such first-level coefficients are represented in a lag-specific [8, 8] empirical transition matrix *B*.

In a second-level regression, we then quantified the evidence that the empirical (lag-specific) transition matrix, *B*, is predicted by the underlying task transition structure (i.e., ‘structural sequences’),

$$\begin{aligned} B=\sum_{r=1}^{4} Z_{r}*T_{r} \end{aligned}$$

, where *B*is the [state, state] empirical transition matrix, *T_r_* is the [state, state] predictor transition matrix (for regressor *r*), and *Z_r_* is the scalar regression coefficient quantifying the evidence that the hypothesized transitions, *T_r_* predict the empirical transitions, *B*. We consider 4 predictor matrices, *T* (i.e., *r* ∈ {1 : 4}): (1) ‘structural sequence’ transitions in the *fwd* direction (transitions corresponding to [*A* → *B* → *C* → *D*] and [*A*^′^ → *B*^′^ → *C*^′^ → *D*^′^] in *T*_1_ are set to 1, all other transitions set to 0), (2) ‘structural sequence’ transitions in the *bwd* direction ([*D* → *C* → *B* → *A*] and [*D*^′^ → *C*^′^ → *B*^′^ → *A*^′^], i.e., *T*_2_ is the transpose of *T*_1_), (3) self-transitions ([8, 8] identity matrix), and (4) a constant matrix.

Sequenceness is defined from the contrast between evidence for sequential replay of task structure in the forward ([*A* → *B* → *C* → *D*] ) vs. backward ([*D* → *C* → *B* → *A*] ) direction (i.e., *Z*_1_−*Z*_2_). Positive sequenceness values indicate replay in a predominantly forward direction. We estimate sequenceness at each state → state transition lag (10 – 600 ms, in 10 ms bins), by repeating the second level regression (above) separately for the empirical transition matrix, *B*, corresponding to each ∆*t*, and report this effect SD-scaled over lags within participants. We previously reported a peak group-level positive sequenceness effect at 40 ms lag ^1^, as in previous MEG replay studies ^8,9^.

Having found maximal evidence for replayed transitions at 40 ms lag in the combined sample of all participants, we identified time points during the rest session where strong reactivation of one stimulus (e.g., A) was followed by strong reactivation of a structurally-adjacent stimulus (e.g., B), with 40 ms lag. Specifically, for each permitted state → state transition in the task design matrix (e.g., *A* → *B*) we quantified a ‘replay evidence’ time course (i.e., *R^A^*^→^*^B^*) as the elementwise product of the individual-state reactivation time courses. For example, for time sample *t* the replay evidence for transition *A* → *B* is

$$\begin{aligned} R_{t}^{\left[ A\to B \right]}=A_{t}*B_{t+40ms} \end{aligned}$$

We then computed a measure of ‘total replay evidence’ as the elementwise sum of all such all individual state → state replay evidence time courses that are permitted by the task transition matrix (*A* → *B*, *B* → *C*, ...). We defined ‘replay onsets’ as time points exceeding the 95^th^ percentile of the participant-specific replay evidence distribution, and preceded by a pre-onset low-probability baseline, identically to Nour et al., (2021).

As detailed in Nour et al., (2021), we epoched the rest data surrounding each replay onset and computed a frequency decomposition (wavelet transformation) in the window -100 to +150 ms with respect to replay onset, for each (non-artefactual) sensor and event. Averaging this estimate over sensors and events resulted in a [time, frequency] matrix for each participant, capturing the typical spectrally-resolved power change at replay onset (**Figure 1A**). For each participant, we defined a separate [time, frequency] matrix using forward and backward task transition matrices (i.e., replay of structurally-adjacent states), and used the average of these matrices for subsequent analyses. For all analyses, individual participant [time, frequency] matrices were SD-scaled within-frequency and across time prior to group level inference.

The above sequenceness and replay onset analyses was conducted in sensor space. To identify neural sources correlating with increased ripple power at identified replay onset times, we next conducted a beamforming analysis on the epoched MEG data, centred on putative replay onsets, as in Nour et al., (2021). Forward models were generated using a single shell using superposition of basis functions that approximately corresponded to the plane tangential to the MEG sensor array. Linearly constrained minimum variance beamforming ^12^ was used to reconstruct the epoched MEG data to a grid in MNI space (grid step = 5 mm).

The sensor covariance matrix for beamforming was estimated using data restricted to 120 - 150 Hz ^9^. All non-artefactual replay epochs were baseline corrected at source level (baseline defined as mean power -100 ms to -10 ms with respect to replay onset). The second level design matrix included separate regressors for main effect of group, subject-specific mean ripple (120 – 150 Hz) power increase at replay onset, and the *group * ripple* interaction. We report whole-brain results for voxels predicted by subject-specific ripple power at onset (0 ± 2.5 ms), and use non-parametric permutation tests on this volume to identify clusters significant at P_FWE_ *<* 0.05 (whole-brain corrected, cluster-defining threshold t = 3, 5000 permutations) (**Figure 1A**).

### **PET kinetic modelling**

As described in Beck et al., (2021), prior to kinetic modelling, all PET scans underwent the same image processing pipeline to correct for subject motion, segment brain tissues and extract [^18^F]GE-179 tracer activity ^2^. NMDAR availability was operationalized as the [^18^F]GE-179 volume of distribution (V_T_). V_T_ captures the ratio between ligand concentration in the brain tissue compartment (comprising specific binding, *C_S_*, non-specific binding, *C_NS_*, and the free tissue radioligand, *C_FT_* ) to total arterial plasma concentration (*C_P_* ) at equilibrium conditions, and is expressed in units of millilitre per cubic centimetre ^13^.

$$\begin{aligned} V_{T}=\frac{\left( C_{S}+C_{NS}+C_{FT} \right)}{C_{P}} \end{aligned}$$

V_T_ was estimated in two ways, which were highly correlated ^2^. In a region of interest (ROI) analysis a single V_T_ measure was derived from [^18^F]GE-179 activity within a single hippocampal mask (e.g., bilateral hippocampus as defined by a probabilistic neuroanatomical atlas ^14^). For this ROI analysis V_T_ was estimated using a 2-tissue compartment modelling method with metabolite-corrected arterial plasma input function ^2,13,15,16^. This approach models the blood to tissue transfer of radioligand and biophysical processes that govern the association between the tracer and the target protein (NMDAR) in the brain (tissue compartment) at equilibrium. The model estimates 4 transfer rate constants: *K*_1_: transfer rate constant of radioligand from tissue plasma into the first (‘non-displaceable’) tissue compartment (comprising free plus non-specifically bound radioligand, *F* +*NS*, which are assumed to be in rapid equilibrium), *k*_2_: transfer in reverse direction (i.e., from ‘non-displaceable’ tissue compartment to plasma), *k*_3_: the transfer rate constant from the first tissue compartment (‘non-displaceable’, comprising *F* +*NS*) to the second tissue compartment (radioligand-receptor specific binding, *S*), and *k*_4_: the rate constant describing the dissociation of the radioligand-receptor complex, and thus a transfer from the ‘specific’ to the ‘non-displaceable’ tissue compartment. *k*_3_ and *k*_4_ thus characterise the dynamics of radioligand-receptor interactions within the brain, in an analogous manner to the *in vitro* association and dissociation rate constants (*k_on_* and *k_off_*, respectively). Specifically, *k*_4_ = *k_off_*, and *k*_3_ = *k_on_*B_avail._*f_ND_*, where *B_avail._* is the density of available receptors in the tissue and *f_ND_* is the fraction of radioligand in the non-displaceable compartment that is freely dissolved in tissue water, i.e., $\frac{F}{F+NS}$ ^13^. A nonlinear estimator was used to identify model parameters at the individual participant level, weighting each data point for the inverse of its error variance (MATLAB *lsqnonlin.m*) ^2^.

The two-tissue compartment model was used to quantify V_T_ as follows:

$$\begin{aligned} V_{T}=\frac{K_{1}}{k_{2}}\left( 1+\frac{k_{3}}{k_{4}} \right) \end{aligned}$$

, where $\frac{k_{3}}{k_{4}}$ is equal to a related ‘binding potential’ measure in PET imaging, *BP_ND_*, which is the ratio at equilibrium of specifically-bound radioligand to that of non-displaceable radioligand in tissue ^13,15^.

V_T_ was also estimated at the individual voxel level, using the Logan graphical approach ^17^. Use of two-tissue compartmental modelling at the individual-voxel level is not routinely performed owing to the lower signal to noise ratio compared to ROI analysis.

The original analysis of the present PET dataset additionally defined a normalized measure of hippocampal [^18^F]GE-179 V_T_ (distribution volume ratio, DVR) at the ROI level, defined as hippocampal [^18^F]GE-179 V_T_ divided by whole-brain [^18^F]GE-179 V_T_ for each participant ^2^. We therefore additionally report ROI analyses using this DVR measure. See **Table 1** for summary group effects pertaining to potential confounding variables for a group difference in V_T_, namely participant movement, ROI volumes and *K*_1_ modelling parameters.

### **Statistical software**

Statistical analysis was performed using MATLAB (Mathworks) 2019a. MEG pre-processing and source reconstruction was performed using MATLAB in conjunction with functions from SPM12 ([https://www.fil.ion.ucl.ac.uk/spm/software/spm12/)](https://www.fil.ion.ucl.ac.uk/spm/software/spm12/), FieldTrip ([http://www. fieldtriptoolbox.org/)](http://www.fieldtriptoolbox.org/), the OHBA Software Library (OSL, including OAT, [https://ohba-an](https://ohba-analysis.github.io/osl-docs/)alysis.[github.io/osl-docs/)](https://ohba-analysis.github.io/osl-docs/) and FMRIB Software Library (FSL, [https://fsl.fmrib.ox.ac.uk/ fsl/fslwiki/)](https://fsl.fmrib.ox.ac.uk/fsl/fslwiki/). For PET analysis, data pre-processing was performed using a combination of SPM12 and FSL functions, as implemented in MIAKAT ^18^.

# Supplementary References

1. Nour MM, Liu Y, Arumuham A, Kurth-Nelson Z, Dolan RJ. Impaired neural replay of inferred relationships in schizophrenia. *Cell*. 2021;184(16). doi:10.1016/j.cell.2021.06.012

2. Beck K, Arumuham A, Veronese M, et al. N-methyl-D-aspartate receptor availability in first-episode psychosis: a PET-MR brain imaging study. *Transl Psychiatry*. 2021;11(1):1-8. doi:10.1038/s41398-021-01540-2

3. First MB, Spitzer RL, Gibbon M, Williams JBW. *Structured Clinical Interview for DSM-IV Axis I Disorders— Patient Edition*. Version 2. New York Biometrics Research Department; 1995.

4. Kay SR, Fiszbein A OL. The Positive and Negative Syndrome Scale (PANSS) for schizophrenia. *Schizophr Bull*. 1987;13(2):261-276. doi:10.1093/schbul/13.2.261

5. Williams JBW, Kobak KA. Development and reliability of a structured interview guide for the Montgomery-Åsberg Depression Rating Scale (SIGMA). *Br J Psychiatry*. 2008;192(1):52-58. doi:10.1192/bjp.bp.106.032532

6. American Psychiatric Association. *Diagnostic and Statistical Manual of Mental Disorders*. 5th ed. American Psychiatric Publishing; 2013.

7. Wechsler D. *Wechsler Test of Adult Reading: WTAR*. The Psychological Corporation; 2001.

8. Kurth-Nelson Z, Economides M, Dolan RJ, Dayan P. Fast Sequences of Non-spatial State Representations in Humans. *Neuron*. 2016;91(1):194-204. doi:10.1016/j.neuron.2016.05.028

9. Liu Y, Dolan RJ, Kurth-Nelson Z, Behrens TEJ. Human Replay Spontaneously Reorganizes Experience. *Cell*. 2019;178(3):640-652. doi:10.1016/j.cell.2019.06.012

10. Wimmer GE, Liu Y, Vehar NNN, Behrens TEJJ, Dolan XRJ, Dolan RJ. Episodic memory retrieval success is supported by rapid replay of episode content. *Nat Neurosci*. 2020;23(8):1025-1033. doi:10.1038/s41593-020-0649-z

11. Liu Y, Dolan RJ, Higgins C, et al. Temporally delayed linear modelling (TDLM) measures replay in both animals and humans. *Elife*. 2021;10:e66917. doi:10.1101/2020.04.30.066407

12. Veen BD Van, Drongelen W Van, Yuchtman M, Suzuki A. Localization of brain electrical activity via linearly constrained minimum variance spatial filtering. IEEE Transactions on. *Biomed Eng (NY)*. 1997;44(9):867-880.

13. Innis RB, Cunningham VJ, Delforge J, et al. Consensus nomenclature for in vivo imaging of reversibly binding radioligands. *J Cereb Blood Flow Metab*. 2007;27(9):1533-1539. doi:10.1038/sj.jcbfm.9600493

14. Hammers A, Allom R, Koepp M, et al. Three-dimensional maximum probability atlas of the human brain, with particular reference to the temporal lobe. *Hum Brain Mapp*. 2003;19(4):224-247.

15. Gunn RN, Slifstein M, Searle GE, Price JC. Quantitative imaging of protein targets in the human brain with PET. *Phys Med Biol*. 2015;60(22):R363-411. doi:10.1088/0031-9155/60/22/R363

16. McGinnity CJ, Hammers A, Riaño Barros DA, et al. Initial evaluation of 18F-GE-179, a putative PET Tracer for activated N-methyl D-aspartate receptors. *J Nucl Med*. 2014;55(3):423-430. doi:10.2967/jnumed.113.130641

17. Logan J, Fowler JS, Volkow ND, et al. Graphical Analysis of Reversible Radioligand Binding from Time—Activity Measurements Applied to [ N - 11 C-Methyl]-(−)-Cocaine PET Studies in Human Subjects. *J Cereb Blood Flow Metab*. 1990;10(5):740-747. doi:10.1038/jcbfm.1990.127

18. Gunn RN, Coello C, Searle G. Molecular Imaging And Kinetic Analysis Toolbox (MIAKAT)- A Quantitative Software Package for the Analysis of PET Neuroimaging Data. *J Nucl Med*. 2016;57:Supplement 2 1928.
